# Comparative analysis of androgen and estrogen receptor mRNA expression in adult mouse hippocampus

**DOI:** 10.1111/jne.70086

**Published:** 2025-08-28

**Authors:** Malte Schöbe, Bianka Brunne, Roland A. Bender

**Affiliations:** ^1^ Institute of Neuroanatomy University Medical Center Hamburg Hamburg Germany

**Keywords:** androgen, estrogen, estrous cycle, GPER1, ZIP9

## Abstract

In hippocampus, androgens and estrogens influence neuronal plasticity via a range of nuclear or membrane‐bound receptors. While much work has focused on determining their functions, a certain vagueness about the cellular expression of established receptors has remained. Moreover, novel candidates, such as the androgen‐responsive zinc‐transporter ZIP9, need to be inserted into the emerging picture. We used highly‐sensitive RNAscope in situ hybridization and quantitative real‐time PCR to examine the cellular and total hippocampal mRNA expression of androgen (AR, ZIP9) and estrogen receptors (ERα, ERβ, GPER1) in adult mouse hippocampus, considering sex and estrous cycle as variables. (1) Androgen receptors are more abundantly expressed than estrogen receptors. (2) AR and ZIP9 mRNA regularly co‐localize in hippocampal neurons, but ZIP9 mRNA is more homogenously distributed and also expressed in astrocytes and microglia. (3) ERα and GPER1 are the predominant estrogen receptors (ERβ mRNA was very low), but exhibit differential expression patterns: GPER1 mRNA is preferentially expressed in glutamatergic neurons, while ERα is specifically expressed in a subpopulation of GABAergic interneurons. Both receptors were barely detectable in astrocytes and microglia. (4) ZIP9 mRNA expression varies during the estrous cycle, being significantly down‐regulated if serum E2 is high, whereas ERα mRNA expression was generally higher in females. We provide a comprehensive cellular and quantitative expression analysis of androgen and estrogen receptors in adult mouse hippocampus, including for the first time mRNA expression data on ZIP9. Our data underline the necessity to consider sex and estrous cycle when studying sex hormone functions.

## 1 INTRODUCTION

Beyond their roles in regulating reproduction, androgens and estrogens are potent neuromodulators influencing a wide variety of brain functions, including processes involved in learning and memory.^1^, ^2^ Both types of sexual steroid hormones exert their functions via various types of steroid receptors, which mediate cellular responses age‐, sex‐, and even cell‐type‐specifically.^3^ For estrogens, functions of two types of receptors, belonging to the nuclear transcription factor family (ERα and ERβ^3^), and of one membrane‐bound, G‐protein‐coupled estrogen receptor (GPER1; previously termed GPR30^4^) are solidly established in the brain. For androgens, a transcriptionally active receptor (termed AR^5^, ^6^, ^7^) has long been considered the only androgen receptor, but in recent years several androgen‐binding proteins, unrelated to AR, have been identified.^8^, ^9^ While for most of these proteins androgen‐related functions in the brain have not yet been identified, the zinc (Zn^2+^) transporter ZIP9 has recently been shown to mediate androgen signaling in the brain.^10^

Knowledge about the cell types expressing these receptors is a precondition for understanding their roles in neuronal networks. However, the data reported in the literature are in part conflicting. Thus, while numerous studies have examined the expression patterns of estrogen receptors in the rodent hippocampus using both immunohistochemistry^11^, ^12^, ^13^, ^14^, ^15^, ^16^, ^17^, ^18^, ^19^, ^20^, ^21^, ^22^, ^23^, ^24^, ^25^ and in situ hybridization (ISH) techniques,^26^, ^27^, ^28^, ^29^ discrepancies remain about which cell types are the main targets of estrogens in the hippocampus. For the androgen receptors, reported expression patterns are congruent at large for AR, indicating predominant AR expression in the CA1 pyramidal layer,^26^, ^30^, ^31^, ^32^, ^33^, ^34^, ^35^, ^36^ but a detailed analysis of the cell types that express AR has not yet been undertaken. Moreover, no information on hippocampal ZIP9 expression is available yet.

To fill these gaps, we here used the highly sensitive RNAscope ISH technique^37^ to define mRNA expression of androgen (AR, ZIP9) and estrogen receptors (ERα, ERβ, GPER1) cell type‐specifically within the adult mouse hippocampus. Because expression of these receptors may vary depending on the sex and the female estrous cycle stage,^15^, ^18^, ^23^ we further applied quantitative real‐time PCR (qRT‐PCR) to determine total hippocampal mRNA expression in adult males and in females during cycle stages, in which serum levels of 17β‐estradiol (E2) are low (metestrus, estrus = “E2 low”) or rising respectively high (diestrus, proestrus = “E2 high”^38^, ^39^; note that Nilsson et al., using highly‐sensitive gas chromatography‐tandem mass spectrometry, found substantial diestrus levels of E2 in mice, but not in rats). We chose the hippocampus for these analyses, because of its outstanding role for learning and memory processes that are influenced sex‐specifically by both estrogens and androgens.^2^, ^40^

## 2 MATERIALS AND METHODS

### 2.1 Animals

A total of 33 young adult mice (C57BL/6J, 10–14 weeks old) were included into these studies; 26 (8 males, 8 “E2 high” females, 10 “E2 low” females) were used for qRT‐PCR, 7 (4 males, 3 “E2 high” [proestrus] females) were used for RNAscope‐ISH. Mice were housed in the animal facility of the University Medical Center Hamburg‐Eppendorf. The animals were kept in a constant day–night cycle (illumination: 6.00 a.m. to 6.00 p.m), with access to food and water ad libitum. All experiments were performed in accordance with institutional guidelines for animal welfare. For the females, estrous cycle stage was determined post mortem (vaginal smears were taken immediately after death), using the staining procedure described by McLean et al.^41^ Tissue was collected at noon time (11 a.m. to 1 p.m.).

### 2.2 RNA isolation, complementary DNA (cDNA) synthesis and quantitative real‐time PCR (qRT‐PCR)

For qRT‐PCR, mice were euthanized with CO_2_/O_2_ (80/20%), followed by CO_2_ (100%). Immediately after death, brains were taken out of the skull, the two hemispheres were split, and basal ganglia, thalamus, and brain stem tissue were removed, thus exposing the hippocampus from medial. Hippocampi were then quickly dissected, using a prominent blood vessel, which characteristically runs above the hippocampus, as an anatomical landmark to distinguish hippocampal from neocortical areas. Finally, white matter remnants were removed, and the hippocampi were deep‐frozen in liquid nitrogen. For RNA isolation, RNeasy Mini Kit (QIAGEN) was used according to the manufacturer's protocol. Briefly, tissue was rapidly homogenized using the Precellys Evolution tissue homogenizer (Bertin Technologies, Montigny‐le‐Bretonneux, France) and DNA was removed using the DNA‐Free DNA removal kit (Thermo Fisher Scientific), according to the manufacturer's instructions. Concentration of total RNA was assessed with DeNovix DS‐11 spectrophotometer (DeNOVIX, Wilmington, DE, USA) and complementary DNA (cDNA) was synthesized from 0.5 μg total RNA using Maxima First Strand cDNA Synthesis kit (ThermoFisher Scientific). Samples were diluted 1/10 in RNase/DNAse free water and processed using the StepOne Real‐Time PCR System (Applied Biosystems) including SYBR Green (Thermo Fisher). PCR was run with the following parameters: 95°C for 3 min → 40 cycles with 95°C for 10 s → annealing temperature (63°C) for 1 min → 95°C for 15 s → 60°C for 1 min. Melting curve increased 0.3°C every 15 s till 95°C. Primers included (FL = fragment length, SR = steroid receptor, HKG = housekeeping gene, Fw = forward, Rv = reverse):

**Table:** 

Gene	Sequence 5′ → 3′	FL (bp)	Marker
GAPDH	Fw: GTCTACTGGTGTCTTCACCACC Rv: GTGAGTTGTCATATTTCTCGTGGTTC	140	HKG
HSP90	Fw: TACTACTACTCGGCTTTCCCGT Rv: TCGAATCTTGTCCAGGGCATC	192	HKG
AR	Fw: CCGTGTGTCTTCTTCTGCAC Rv: CTTCCACCTACTTCCCTTACC	189	SR
ERα	Fw: CCAGGCTTTGTGGATTTGAC Rv: TGGTTCCTGTCCAAGAGCAA	149	SR
ERβ	Fw: AATGGTGAAGTGTGGCTCCC Rv: ACTTCTCTTGGCCTTGCCG	106	SR
GPER1	Fw: AGGTACCCAGAGAGTGAGC Rv: GGTGGTTTGGGTTGGGTTTG	175	SR
ZIP9	Fw: GCTGATGGTGTTGCTTTGGG Rv: GATTCGATTCCGCTCTAAGCC	150	SR

Specificity of all primers was validated by sequencing of the PCR product; the melting curve for the sequenced reaction was used as reference in all following PCRs.

### 2.3 RNAscope in situ hybridization (ISH)

For RNAscope ISH, mice were euthanized as described above, then transcardially perfused post mortem (immediately after death), using ice‐cold PBS followed by 4% paraformaldehyde solution (PFA, in PBS). Subsequently, the brains were removed from the skull, postfixed for 4 h in 4% PFA, cryoprotected with 25% sucrose/PBS for >48 h, and deep frozen in −50°C isopentane. Frozen brains were sectioned coronally (14 μm) and sections including dorsal hippocampus (between Bregma −1.7 and −2.4 mm^42^) were transferred to SuperFrost glass slides (one section each slide), which were stored at −80°C until further use. For all steps above, RNAse‐free solutions and tools were used. RNAscope ISH was performed according to a protocol for fixed‐frozen tissue using the Advanced Cell Diagnostics (ACD) RNAscope Multiplex Fluorescent Reagent Kit V2 (Cat. No. 323100). Sections were air‐dried and processed for mRNA detection of one of the receptors (ERα, ERβ, GPER1, AR, or ZIP9) in combination with a cell type‐specific marker, that is, Ca^2+^/calmodulin‐dependent protein kinase II (CamKII), glutamate decarboxylase 65 (GAD65), glial fibrillary acidic protein (GFAP), or purinergic receptor P2Y12 mRNA, indicative of glutamatergic projection neurons, GABAergic interneurons, astrocytes, and microglia, respectively. Sections were counterstained with the nuclear marker 4,6‐diamidino‐2‐phenylindol (DAPI, Sigma). Sections from at least 2 female and 2 male brains were processed for each receptor/cell marker combination (precise numbers of analyzed sections resp. mice are provided in the figure legends). Further, to check for co‐expression of ZIP9 and AR mRNA, differentially tagged probes (C1 and C3, respectively) were co‐applied in some experiments. The following mouse‐specific sequences were used for mRNA detection (all from ACD Bio):

**Table:** 

Target mRNA	Sequence	ACD Cat. no.
ZIP9	RNAscope™ Probe‐Mm‐Slc39a9‐C1	588061‐C1
AR	RNAscope™ Probe‐Mm‐Ar‐C3	316991‐C3
ERα	RNAscope™ Probe‐Mm‐Esr1‐C3	478201‐C3
ERβ	RNAscope™ Probe‐Mm‐Esr2‐C3	316121‐C3
GPER1	RNAscope™ Probe‐Mm‐Gper1‐C3	475251‐C3
CamKII	RNAscope™ Probe‐Mm‐Camk2a‐C2	445231‐C2
GAD65	RNAscope™ Probe‐Mm‐Gad2‐C2	439371‐C2
GFAP	RNAscope™ Probe‐Mm‐Gfap‐C2	313211‐C2
P2Y12	RNAscope™ Probe‐Mm‐P2ry12‐C2	317601‐C2

### 2.4 Analyses

**FIGURE 1:**
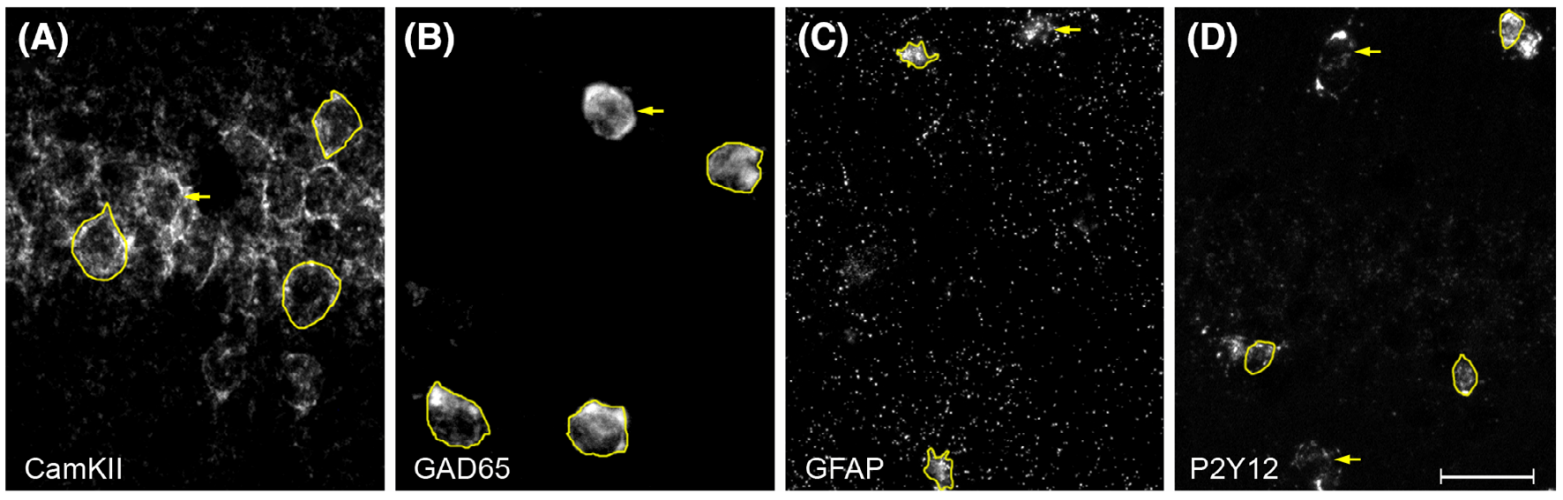
Selection of regions of interest (ROI) for RNAscope analysis. Photographs illustrate the selection of regions of interest (ROI) for subsequent analysis of receptor mRNA in cells expressing CamKII (A), GAD65 (B), GFAP (C), and P2Y12 (D). ROIs were manually defined (yellow circles) using confocal pictures showing only the cell marker mRNA. Cells were selected for analysis if cell type‐specific morphology was clearly recognizable, that is: round or oval‐shaped somata of 10–15 μm Ø, indicative of CamKII‐positive projection neurons (A^104^) and GAD65‐positive interneurons (B^91^), star‐shaped cells with round or oval somata of 5–10 μm Ø, indicative of GFAP‐positive protoplasmic astrocytes (C^105^) and oval‐shaped somata of 8–10 μm Ø with fine extensions, indicative of P2Y12‐positive microglia (D^106^). As cell marker mRNA signal does not fully denote cell boundaries, ROIs were confined to the labeled parts of the soma and did not include appendages (i.e., neuronal dendrites, astrocytic or microglia processes). Arrows indicate unmarked cell somata, which are also suitable for analysis. Scale bar: 20 μm.

For RNAscope ISH analysis, representative pictures were taken from CA1, CA3, and the dentate gyrus (DG) in the dorsal hippocampus using a Leica SP5 confocal microscope (63 × 1.4 objective). Optimal conditions for receptor mRNA recording were determined in pilot experiments and then continuously applied throughout the study (AR, ERα: 10% laser intensity [655 nM], 10% gain; GPER1: 10% laser intensity [655 nM], 15% gain; ERβ: 14% laser intensity [655 nM], 15% gain; ZIP9: 5% laser intensity [561 nM], 10% gain). Conditions for cell marker mRNA scanning were determined accordingly. After capturing the confocal pictures, the channels depicting the signal for receptor mRNA (C1, C3), cell‐type marker mRNA (C2) or DAPI nuclear stain (C4) were separated. An investigator blinded to the receptor signal (i.e., unaware of the data from channels C1 and C3) then defined the regions of interest (ROI) by manually outlining the cell somata of individual projection neurons, GABAergic interneurons, astrocytes, or microglia using the cell marker channel C2 (see Figure 1 for details). Additionally, a cell‐free area was defined using the DAPI channel (C4). Subsequently, a second investigator used the receptor channels to determine pixel intensity within the ROI (=raw receptor signal). To obtain the final receptor mRNA signal, the background signal, as determined in the cell‐free area, was subtracted from the raw receptor signal within the ROI. Resulting data are presented as arbitrary units (AU).

**FIGURE 2:**
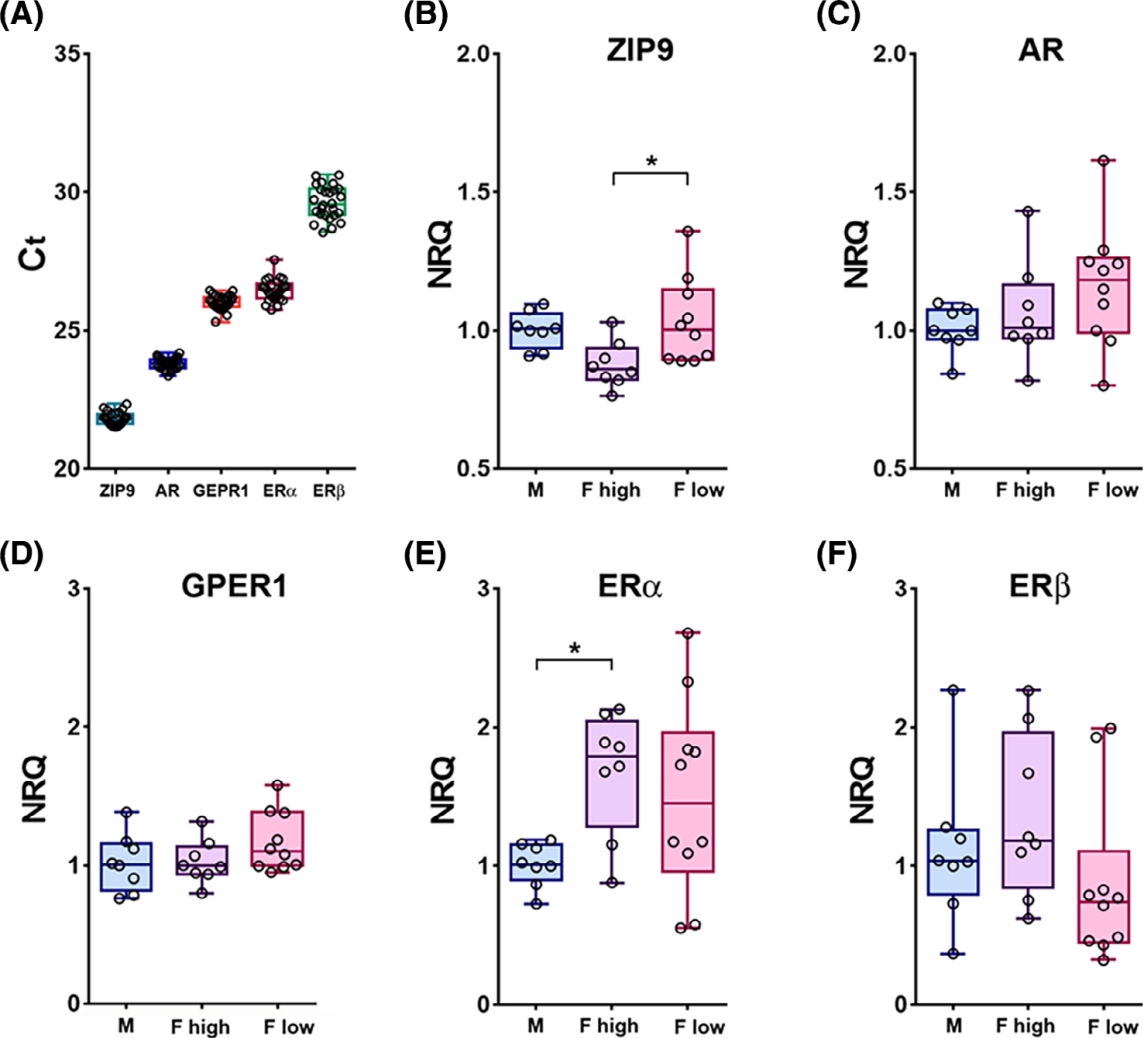
Total mRNA expression levels of androgen and estrogen receptors in adult mouse hippocampus. (A) Cycle thresholds (*C*_*t*_) were significantly different between receptor mRNA types (One‐Way‐ANOVA, *F* = 1652, *p* < .001, *n* = 26 each receptor; Tukey: *p* < .001 for all pairings), indicating a markedly stronger mRNA expression of androgen receptors (*C*_*t*_, ZIP9: 21.8 ± 0.1, AR: 23.8 ± 0.1) compared with estrogen receptors (*C*_*t*_, GPER1: 26.0 ± 0.1, ERα: 26.5 ± 0.1, ERβ: 29.6 ± 0.1). (B–F) Comparison of hippocampal mRNA expression levels between males (M, blue bars, *n* = 8), “E2 high” females (F high, violet bars, *n* = 8) and “E2 low” females (F low, pink bars, *n* = 10) did not reveal significant differences for AR (C, M: 1.00 ± 0.03, F high: 1.06 ± 0.06, F low: 1.16 ± 0.07; One‐Way‐ANOVA, F: 1.89, *p* = .17), GPER1 (D, M: 1.00 ± 0.07, F high: 1.03 ± 0.06, F low: 1.17 ± 0.07; Kruskal–Wallis Statistics: 2.86, *p* = .24) and ERβ (F, M: 1.00 ± 0.19, F high: 1.36 ± 0.21, F low: 0.87 ± 0.19; Kruskal–Wallis Statistics: 4.27, *p* = .12). However, ZIP9 mRNA expression was significantly lower in the “E2 high” compared with the “E2 low” cycle stage, suggesting down‐regulation of expression by fluctuating sex hormones (B, M: 1.00 ± 0.02, F high: 0.88 ± 0.03, F low: 1.03 ± 0.05; One‐Way‐ANOVA, F: 4.45, *p* = .02; Tukey: M vs. F high, *p* = .07; M vs. F low, *p* = .59; F high vs. F low, *p* = .03). Moreover, analysis of ERα mRNA levels indicated a stronger expression in females compared with males, particularly if E2 is high (E, M: 1.00 ± 0.05, F high: 1.68 ± 0.16, F low: 1.50 ± 0.22; One‐Way‐ANOVA, F: 3.63, *p* = .04; Tukey: M vs. F high, *p* = .04; M vs. F low, *p* = .13; F high vs. F low, *p* = .74). Asterisks indicate levels of statistical significance: *p < 0.05, **p < 0.01, ***p < 0.001.

For qRT‐PCRs, 3 runs were executed for each receptor mRNA, each comprising a mixture of male, female “E2 high” and female “E2 low” samples. Datasets for every target (i.e., receptor mRNA) are based on the exactly same group of cDNA samples. Data analysis was performed with Qbase+ software (Biogazelle), generating Normalized Relative Quantity (NRQ) values, which were calculated from the threshold cycle (*C*_*t*_) of the gene of interest (*goi*) and of multiple reference genes (*f*) according to Hellemans et al.^43^: NRQ=E_goi^∆Ctgoi/√f&∏0fEref0∆Ct,ref0. Note: for comparison of sex and cycle stage (Figure 2B–F), qRT‐PCR data from females (“E2 high” and “E2 low”) were scaled to the mean of the male data within each run.

### 2.5 Statistics

Statistical analyses were performed using Prism software (GraphPad 6). For all data sets, Kolmogorov–Smirnov test was applied to analyze data for normal distribution. For qRT‐PCR, data were subsequently analyzed for statistical differences of *C*_*t*_ values and effects of sex or estrous cycle on individual receptor mRNA expression using One‐Way‐ANOVA, if data were normally distributed, or Kruskal–Wallis test, if data were not normally distributed (*n* = animals). Post hoc analyses were performed with Tukey's or Dunn's multiple comparison tests, respectively. The RNAscope data were statistically analyzed using Kruskal–Wallis tests (as individual data points were mostly not normally distributed) to determine subregional differences (CA1, CA3, and DG) of receptor expression. Additionally, Two‐Way‐ANOVA using “sex” and “cell type” as fixed factors and “receptor mRNA expression” as dependent variable was applied to identify differences between sexes and variances among cell types. Post hoc analyses were performed using Dunn's (Kruskal–Wallis) or Bonferroni's (Two‐Way ANOVA) multiple comparison tests. Note: Data from individual cells (*n* = cells), not means from animals, were used for these analyses.

Data are graphically illustrated with box‐and‐whisker plots (min‐to‐max, showing the median and all data points; qRT‐PCR, RNAscope) or with bar plots, showing mean and standard error of the mean (SEM; male–female comparison of RNAscope). Data presentation in the text is mostly descriptive. Statistical details (type of test, test statistics, mean ± SEM, *p*‐values) are provided in the figure legends. Levels of significance are two‐tailed and set to *p* < .05 (*), *p* < .01 (**), or *p* < .001 (***).

## 3 RESULTS

### 3.1 Total mRNA expression of sex hormone receptors in adult mouse hippocampus

In order to generally estimate mRNA expression levels of androgen and estrogen receptors in the mouse hippocampus and to identify potential sex differences, we first performed qRT‐PCR analyses with hippocampal tissue from adult male mice and from female mice that were in a stage of the estrous cycle during which E2 serum levels are either high or rising (“E2 high”) or low (“E2 low”^38^, ^39^). We first noted that the cycle threshold (*C*_*t*_) differed significantly between receptors (Figure 2A), being lowest for ZIP9 mRNA (21.8 ± 0.1) and highest for ERβ mRNA (29.6 ± 0.1). As *C*_*t*_ values increase on an exponential scale, this difference indicates a much stronger (presumably >100×) expression of ZIP9 compared with ERβ mRNA. Cycle thresholds for the other receptor mRNAs were intermediate to these extremes (AR: 23.8 ± 0.1, GPER1: 26.0 ± 0.1, ERα: 26.5 ± 0.1; *n* = 26 for each receptor). But overall, a stronger mRNA expression of androgen receptors, if compared with estrogen receptors, was noted both in males and females.

We next compared hippocampal expression levels of individual receptor mRNAs with respect to sex and estrous cycle stage. In these analyses, we did not observe significant differences between groups for AR (Figure 2C), GPER1 (Figure 2D), and ERβ (Figure 2F). However, ZIP9 mRNA levels were found to be significantly lower in “E2 high” if compared with “E2 low” females, suggesting that ZIP9 is subject to regulation by sex hormones which fluctuate during the estrous cycle (Figure 2B). In addition, ERα mRNA was significantly enhanced in “E2 high” females if compared with males, but not if compared with “E2 low” females. Although regulation by sex hormones could here also play a role, the absence of a distinct cycle effect suggests that factors unrelated to the estrous cycle contribute to a sex‐specifically enhanced ERα expression in the female mouse hippocampus.^21^

### 3.2 Cell type‐specific mRNA expression of sex hormone receptors in adult mouse hippocampus

#### 3.2.1 Androgen receptors

**FIGURE 3:**
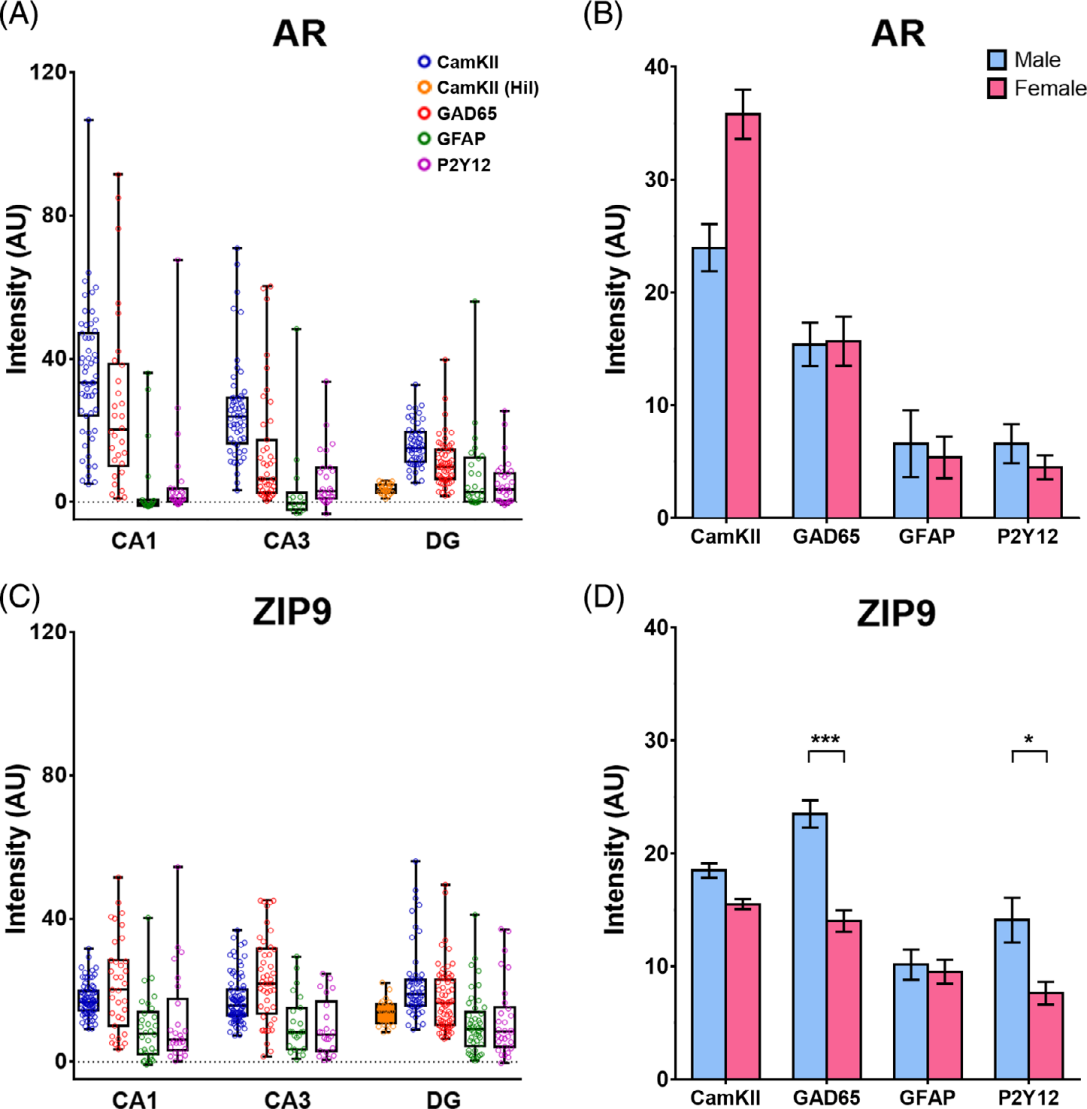
Quantitative analyses of mRNA expression of androgen receptors AR and ZIP9 in adult mouse hippocampus. (A) Box‐and‐Whisker plots (min‐to‐max, showing the median and all data points) illustrate signal intensities indicative of AR mRNA expression in cells co‐expressing Ca^2+^/calmodulin‐dependent protein kinase II (CamKII) in major glutamatergic projection neurons of the hippocampus, that is, pyramidal cells (blue circles) of CA1 and CA3, as well as granule cells (blue circles) and mossy cells (CamKII Hil, orange circles) of dentate gyrus (DG). In addition, AR mRNA expression in cells co‐expressing glutamate decarboxylase 65 (GAD65, i.e., GABAergic interneurons; red circles), glial fibrillary acidic protein (GFAP, i.e., astrocytes; green circles) or purinergic receptor P2Y12 mRNA (microglia; purple circles) is indicated. Note that AR mRNA shows regional differences: Expression is strongest in pyramidal cells (35.4 ± 2.5, *n* = 55) and GABAergic interneurons (27.2 ± 4.4, *n* = 30) of CA1, but significantly decreases towards CA3 (pyramidal cells: 25.5 ± 1.9, *n* = 55; interneurons: 13.7 ± 2.5, *n* = 43) and DG (granule cells: 15.9 ± 0.8, *n* = 55; interneurons: 11.0 ± 0.9, *n* = 59; Kruskal–Wallis Statistics [CamKII, subregion]: 45.72, *p* < .001; Dunn: *p* < .001 for all pairings; Kruskal–Wallis Statistics [GAD65, subregion]: 13.77, *p* = .001; Dunn: CA1 vs. CA3, *p* = .009; CA3 vs. DG, *p* > .99). In contrast, expression in astrocytes (CA1: 4.5 ± 2.6, *n* = 19; CA3: 4.0 ± 3.6, *n* = 14; DG: 7.6 ± 2.3, *n* = 26) and microglia (CA1: 5.6 ± 2.7, *n* = 27; CA3: 6.3 ± 1.6, *n* = 26; DG: 5.1 ± 1.1, *n* = 31) was generally low in hippocampal subfields. In hilar mossy cells, AR mRNA was also virtually undetectable (3.6 ± 0.3, *n* = 29). (B) Two‐Way‐ANOVA of AR mRNA expression with fixed factors “sex” (male: Blue bars; female: Red bars) and “cell type” indicated general expression differences between cell types (Two‐way‐ANOVA [cell type]: *F*(3, 376) = 55.72, *p* < .001; Bonferroni: *p* < .001 for all combinations, except for GFAP vs. P2Y12: *p* > .99), but no effect of sex (Two‐way‐ANOVA [sex]: *F*(1, 376) = 1.891, *p* = .17). (C) For ZIP9 mRNA, a more homogenous distribution was observed, lacking significant subregional differences. Robust expression was also seen in pyramidal cells (CA1: 17.5 ± 0.5, *n* = 77; CA3: 17.3 ± 0.7, *n* = 85) and in interneurons (CA1: 21.5 ± 2.2, *n* = 35; CA3: 23.0 ± 1.8, *n* = 45; DG: 17.4 ± 1.1, *n* = 68), but astrocytes (CA1: 9.1 ± 1.7, *n* = 30; CA3: 10.5 ± 1.7, *n* = 23; DG: 10.5 ± 1.2, *n* = 46) and microglia (CA1: 11.6 ± 2.5, *n* = 26; CA3: 9.6 ± 1.7, *n* = 22; DG: 11.6 ± 1.9, *n* = 30) also expressed substantial levels of ZIP9 mRNA. Moreover, both glutamatergic cell types of the DG, the granule cells (21.7 ± 1.4, *n* = 54) and the mossy cells (13.8 ± 0.7, *n* = 26), expressed ZIP9 mRNA markedly. (D) Two‐Way‐ANOVA of ZIP9 mRNA with fixed factors “sex” (male: Blue bars; female: Red bars) and “cell type” indicated both an effect of sex (Two‐way‐ANOVA [sex]: *F*(1, 472) = 35.6, *p* < .001) and of cell type (Two‐way‐ANOVA [cell type]: *F*(3, 472) = 29.39, *p* < .001). Bonferroni post‐hoc analyses revealed a significant reduction of ZIP9 mRNA in the female sections, specifically in interneurons and in microglia (CamKII: Male 18.5 ± 0.6, *n* = 102, vs. female 15.5 ± 0.4, *n* = 60, *p* = .12; GAD65: Male 23.5 ± 1.2, *n* = 94, vs. female 14.0 ± 1.0, *n* = 54, *p* < .001; GFAP: Male 10.2 ± 1.3, *n* = 40, vs. female 9.5 ± 1.1, *n* = 52, *p* > .99; P2Y12: Male 14.1 ± 2.0, *n* = 41, vs. female 7.6 ± 1.0, *n* = 37, *p* = .03). Further: Significant differences of expression were detected between cell types (Bonferroni: CamKII vs. GAD65: *p* = .03, all other combinations: *p* < .001), except for astrocytes and microglia (*p* > .99). For optical clarity, not all significant differences are illustrated in the figure. Asterisks indicate levels of statistical significance: *p < 0.05, **p < 0.01, ***p < 0.001.

**FIGURE 4:**
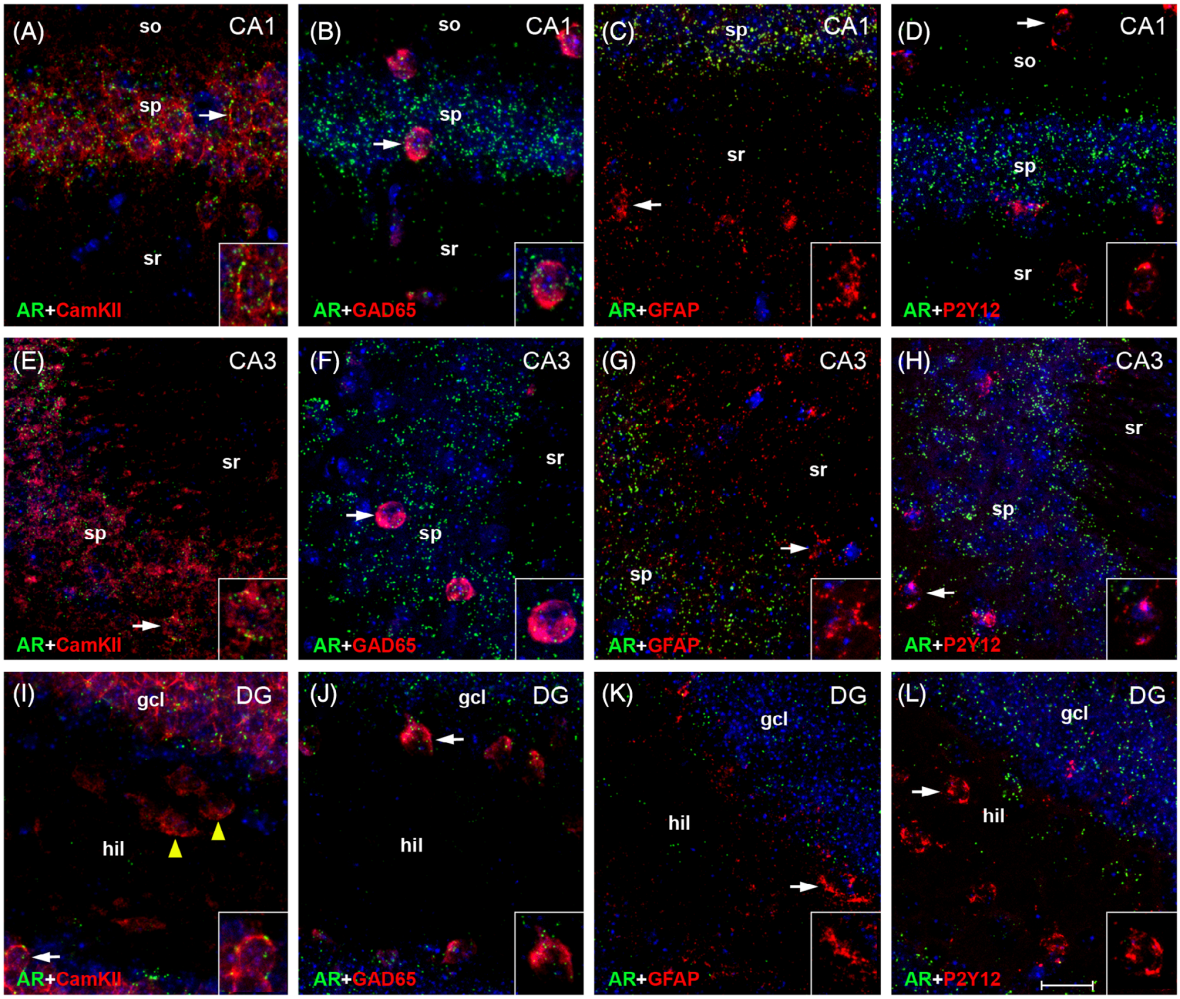
Cell type‐specific expression of AR mRNA in adult mouse hippocampus. Representative photographs illustrate the expression of AR mRNA in hippocampal CA1 (A–D), CA3 (E–H), and dentate gyrus (DG, I–L). Insets show higher magnification views of characteristic cells (indicated by arrows). Note that the signal is comparably strong in Ca^2+^/calmodulin‐dependent protein kinase II (CamKII)‐expressing pyramidal cells of CA1 (A) and CA3 (E). It is further regularly observed in glutamate decarboxylase 65 (GAD65)‐expressing interneurons in CA1 (B, arrows), CA3 (F, arrows) and DG (J, arrows), but barely detectable in glial fibrillary acidic protein (GFAP)‐expressing astrocytes (C, G, K) or in P2Y12‐expressing microglia (D, H, L). In DG, AR mRNA expression is comparably low (relative to pyramidal cells) in CamKII‐expressing granule cells (I, arrow) and virtually absent in CamKII‐expressing hilar cells (presumably mossy cells; I, arrowheads). gcl, granule cell layer; hil, hilus; so, stratum oriens; sp, stratum pyramidale; sr, stratum radiatum. Scale bar: 20 μm (Inset: 10 μm).

**FIGURE 5:**
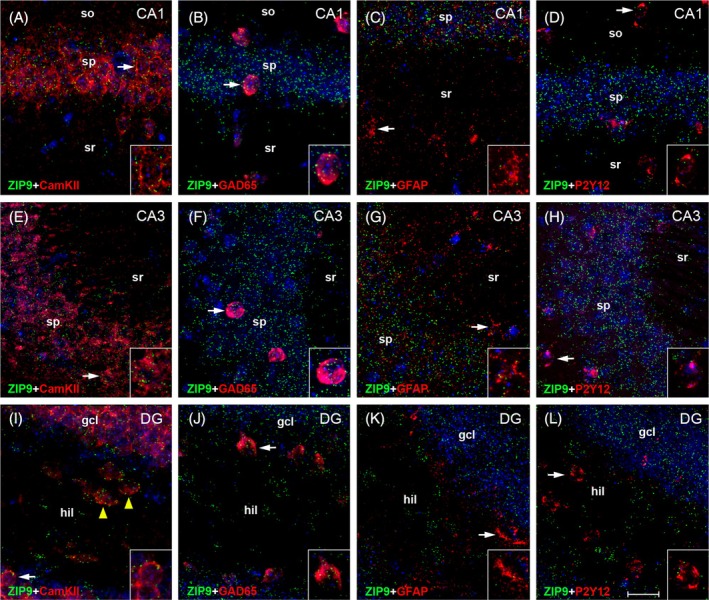
Cell type‐specific expression of ZIP9 mRNA in adult mouse hippocampus. Representative photographs illustrate the expression of ZIP9 mRNA in hippocampal CA1 (A–D), CA3 (E–H), and dentate gyrus (DG, I–L). Insets show higher magnification views of characteristic cells (indicated by arrows). Note that views are identical to those in Figure 4, as pictures were taken from sections, which were co‐labeled for AR and ZIP9 mRNA to examine co‐expression of both androgen receptors. Indeed, AR and ZIP9 mRNA regularly co‐localize in Ca^2+^/calmodulin‐dependent protein kinase II (CamKII)‐expressing pyramidal and granule cells, and in glutamate decarboxylase 65 (GAD65)‐expressing interneurons (compare Figures 4 and 5A,B,E,F,I,J). However, contrary to AR, ZIP9 mRNA is also markedly expressed in glial fibrillary acidic protein (GFAP)‐expressing astrocytes (C, G, K) and in P2Y12‐expressing microglia (D, H, L). Moreover, while CamKII‐expressing cells in the DG hilus do not express AR mRNA, substantial ZIP9 mRNA signal was detected also in these cells (compare Figures 4I and 5I, arrowheads). Thus, compared with AR, the expression of ZIP9 mRNA is more ubiquitous and involves astrocytes, microglia, and (presumed) hilar mossy cells. gcl, granule cell layer; hil, hilus; so, stratum oriens; sp, stratum pyramidale; sr, stratum radiatum. Scale bar: 20 μm (Inset: 10 μm).

RNAscope ISH revealed abundant hippocampal expression of both AR and ZIP9 mRNA (Figures 3, 4, 5), but regional and cell type‐related differences applied. For AR mRNA, the most robust expression was found in CamKII‐positive pyramidal cells of CA1 (Figures 3A and 4A). Lower levels were observed in these cells in CA3 (Figures 3A and 4E) and in the granule cells of DG (Figures 3A and 4I). In CamKII‐positive neurons of the DG hilus (presumably excitatory mossy cells,^44^) AR mRNA was virtually undetectable (Figure 3A, arrowheads in Figure 4I). Substantial expression of AR mRNA was further observed in GAD65‐positive interneurons, but also with a decreasing tendency within hippocampal subfields (Figures 3A and 4B,F,J). In contrast, AR mRNA signal in GFAP‐positive astrocytes (Figures 3A and 4C,G,K) and in P2Y12‐positive microglia (Figures 3A and 4D,H,K) was low in all hippocampal subregions.

ZIP9 mRNA did regularly co‐localize with AR mRNA in CamKII‐ (Figures 3C and 5A,E,I) and GAD65‐positive neurons (Figures 3C and 5B,F,J; note that views in Figures 4 and 5 are identical), but overall ZIP9 mRNA expression was more homogenous in hippocampal subfields and did not show significant subregional differences. Substantial ZIP9 mRNA signal was also detected in astrocytes (Figures 3C and 5C,G,K) and microglia (Figures 3C and 5D,H,K), although with lower intensity, when compared with projection neurons and interneurons (Figure 3D). Remarkably, substantial ZIP9 mRNA signal was also detected in CamKII‐positive neurons of the hilus (Figures 3C and 5I arrowheads).

A comparison of cellular expression data from male and female sections using Two‐Way‐ANOVA did not reveal significant differences between the sexes for AR mRNA expression (Figure 3B), but indicated that ZIP9 mRNA expression was down‐regulated in the sections from females, concordant with the findings from the qRT‐PCR analyses (Figure 2B; note: only sections from females in proestrus were used for RNAscope analysis). Cell‐type specific analyses further suggested that this reduction mainly involves expression in interneurons and microglia (Figure 3D).

#### 3.2.2 Estrogen receptors

**FIGURE 6:**
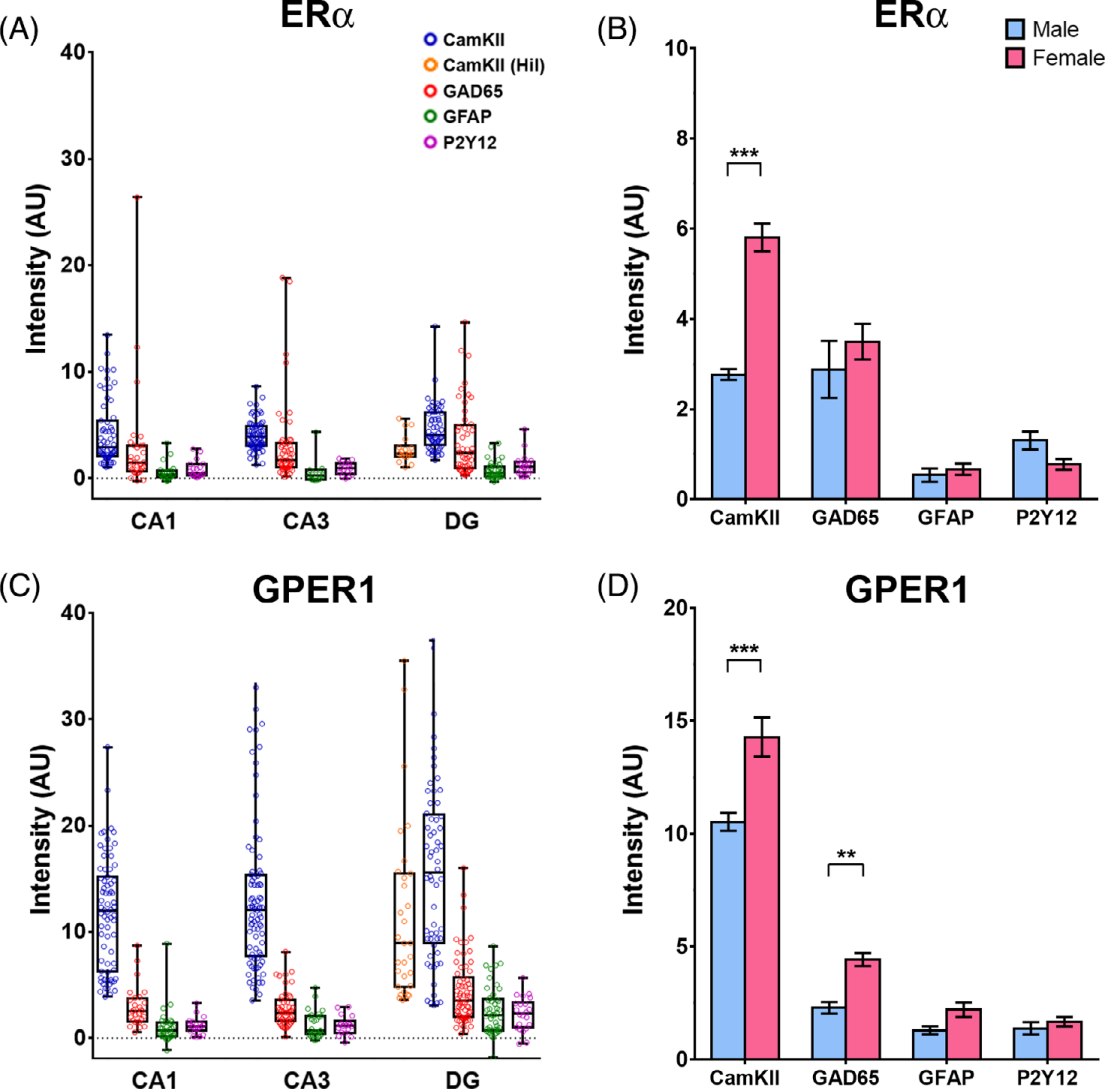
Quantitative analyses of mRNA expression of estrogen receptors ERα and GPER1 in adult mouse hippocampus. (A) Box‐and‐whisker plots (min‐to‐max, showing the median and all data points) illustrate signal intensities indicative of ERα mRNA expression in cells co‐expressing Ca^2+^/calmodulin‐dependent protein kinase II (CamKII) in major glutamatergic neurons of the hippocampus, that is, pyramidal cells (blue circles) of CA1 and CA3, as well as granule cells (blue circles) and mossy cells (CamKII Hil, orange circles) of dentate gyrus (DG). In addition, ERα mRNA expression in cells co‐expressing glutamate decarboxylase 65 (GAD65, i.e., GABAergic interneurons; red circles), glial fibrillary acidic protein (GFAP, i.e., astrocytes; green circles) or purinergic receptor P2Y12 mRNA (microglia; purple circles) is indicated. In general, ERα mRNA expression was low (if compared with androgen receptors) in all examined cellular subtypes. Subregional differences were not detected. Basal mRNA levels were detected in pyramidal cells (CA1: 4.2 ± 0.4, *n* = 59; CA3: 4.0 ± 0.2, *n* = 71) and in granule cells (4.7 ± 0.3, *n* = 57) and hilar mossy cells (2.7 ± 1.3, *n* = 18) of DG. In contrast, ERα signal was virtually undetectable in astrocytes (CA1: 0.6 ± 0.2, *n* = 23; CA3: 0.5 ± 0.3, *n* = 14; DG: 0.7 ± 0.1, *n* = 40) and microglia (CA1: 0.8 ± 0.2, *n* = 19; CA3: 0.9 ± 0.2, *n* = 16; DG: 1.2 ± 0.2, *n* = 20). ERα expression in interneurons was on average also low (CA1: 3.0 ± 0.9, *n* = 30; CA3: 3.1 ± 0.5, *n* = 51; DG: 3.5 ± 0.5, *n* = 49), but some GAD65‐positive cells showed remarkably strong ERα signal, indicating that a subpopulation of GABAergic interneurons expresses ERα robustly (see also Figure 7B,F,J). (B) Two‐Way‐ANOVA of ERα mRNA with fixed factors “sex” (male: Blue bars; female: Red bars) and “cell type” indicated both an effect of sex (Two‐way‐ANOVA [sex]: *F*(1, 383) = 8.0, *p* = .005) and of cell type (Two‐way‐ANOVA [cell type]: *F*(3, 383) = 38.86, *p* < .001). Bonferroni post‐hoc analyses revealed a significantly higher ERα mRNA expression in the female sections, specifically in projection neurons (CamKII: Male 2.8 ± 0.1, *n* = 72, vs. female 5.8 ± 0.3, *n* = 58, *p* < .001; GAD65: Male 2.9 ± 0.6, *n* = 57, vs. female 3.5 ± 0.4, *n* = 73, *p* = .7; GFAP: Male 0.5 ± 0.1, *n* = 23, vs. female 0.7 ± 0.1, *n* = 25, *p* > .99; P2Y12: Male 1.3 ± 0.2, *n* = 41, vs. female 0.8 ± 0.1, *n* = 29, *p* > .99). Generally, ERα mRNA expression was stronger in neurons if compared with astrocytes or microglia, in which barely any signal was detectable (Bonferroni: CamKII vs. GAD65: *P* = .03, GFAP vs. P2Y12: *p* > .99, all other combinations: *p* < .001). (C) Contrary to ERα, GPER1 mRNA was prominently expressed in CamKII‐expressing pyramidal cells (CA1: 11.7 ± 0.6, *n* = 72; CA3: 13.2 ± 0.8, *n* = 85), granule cells (15.6 ± 1.1, *n* = 60) and hilar mossy cells (11.3 ± 1.5, *n* = 31), but barely detectable in interneurons (CA1: 2.9 ± 0.4, *n* = 28; CA3: 2.8 ± 0.2, *n* = 47; DG: 4.4 ± 0.4, *n* = 66), astrocytes (CA1: 1.0 ± 0.3, *n* = 34; CA3: 1.3 ± 0.2, *n* = 28; DG: 2.6 ± 0.3, *n* = 46) and microglia (CA1: 1.2 ± 0.2, *n* = 19; CA3: 1.2 ± 0.2, *n* = 18; DG: 2.3 ± 0.3, *n* = 22). Significant subregional differences were not observed. (D) Two‐Way‐ANOVA of GPER1 mRNA with fixed factors “sex” (male: Blue bars; female: Red bars) and “cell type” indicated both an effect of sex (Two‐way‐ANOVA [sex]: *F*(1, 455) = 16.87, *p* < .001) and of cell type (Two‐way‐ANOVA [cell type]: *F*(3, 455) = 215.5, *p* < .001). Bonferroni post‐hoc analyses revealed a significantly higher GPER1 mRNA expression in the female sections, specifically in projection neurons (CamKII: Male 10.5 ± 0.4, *n* = 75, vs. female 14.3 ± 0.9, *n* = 82, *p* < .001) and interneurons (GAD65: Male 2.3 ± 0.3, *n* = 58, vs. female 4.4 ± 0.3, *n* = 83, *p* < .01), whereas no significant difference was found for astrocytes (GFAP: Male 1.3 ± 0.2, *n* = 55, vs. female 2.2 ± 0.3, *n* = 53, *p* = .92) and microglia (P2Y12: Male 1.4 ± 0.3, *n* = 13, vs. female 1.7 ± 0.2, *n* = 44, *p* > .99). Generally, GPER1 mRNA expression was markedly stronger in projection neurons if compared with all other cell types (CamKII vs. GAD65, GFAP or P2Y12, *p* < .001 each). Further, expression was stronger in interneurons if compared with astrocytes (GAD65 vs. GFAP, *p* = .002) and to microglia (GAD65 vs. P2Y12, *p* = .01), whereas no significant difference was observed between astrocytes and microglia (GFAP vs. P2Y12: *p* > .99). For optical clarity, not all significant differences are illustrated in the figure. Asterisks indicate levels of statistical significance: *p < 0.05, **p < 0.01, ***p < 0.001.

**FIGURE 7:**
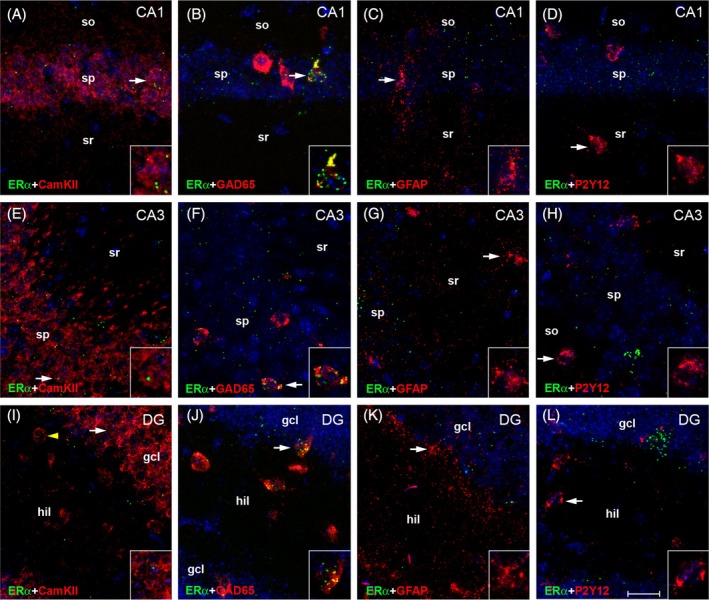
Cell type‐specific expression of ERα mRNA in adult mouse hippocampus. Representative photographs illustrate expression of ERα mRNA in hippocampal CA1 (A–D), CA3 (E–H), and dentate gyrus (DG, I–L). Insets show higher magnification views of characteristic cells (indicated by arrows). Low ERα mRNA signal was detected in Ca^2+^/calmodulin‐dependent protein kinase II (CamKII)‐expressing pyramidal cells of CA1 (A) and CA3 (E), as well as in granule cells (I) and hilar cells (I, arrowhead) in DG. Barely any signal was detectable in glial fibrillary acidic protein (GFAP)‐expressing astrocytes (C, G, K) and in P2Y12‐expressing microglia (D, H, L). In contrast, distinct clusters of signal were observed in some, but not all, glutamate decarboxylase 65 (GAD65)‐expressing interneurons (B, F, J), suggesting that ERα mRNA is preferentially expressed in a subpopulation of GABAergic interneurons in adult mouse hippocampus. gcl, granule cell layer; hil, hilus; so, stratum oriens; sp, stratum pyramidale; sr, stratum radiatum. Scale bar: 20 μm (Inset: 10 μm).

**FIGURE 8:**
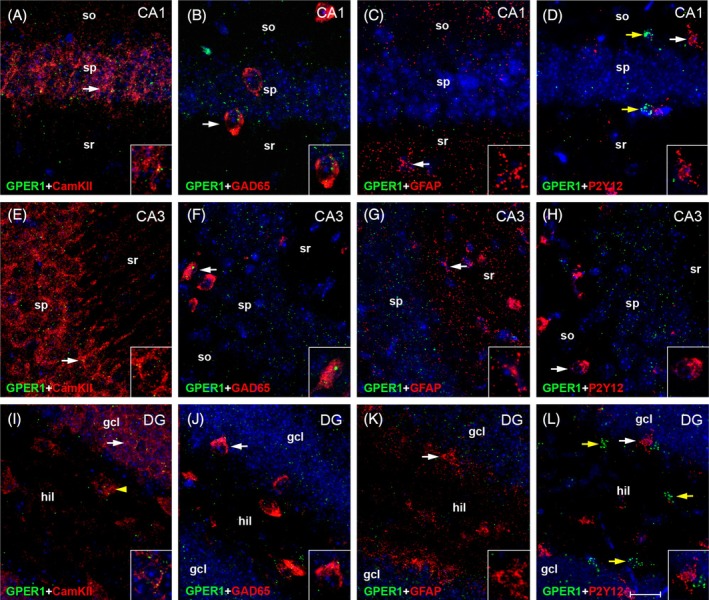
Cell type‐specific expression of GPER1 mRNA in adult mouse hippocampus. Representative photographs illustrate the expression of GPER1 mRNA in hippocampal CA1 (A–D), CA3 (E–H), and dentate gyrus (DG, I–L). Insets show higher magnification views of characteristic cells (indicated by arrows). Moderate GPER1 mRNA signal was detected in Ca^2+^/calmodulin‐dependent protein kinase II (CamKII)‐expressing pyramidal cells of CA1 (A) and CA3 (E), and in DG granule cells (I) and (presumed) mossy cells (I, arrowhead). GPER1 mRNA signal was occasionally also observed in glutamate decarboxylase 65 (GAD65)‐expressing interneurons (B, F, J), but was virtually absent in glial fibrillary acidic protein (GFAP)‐expressing astrocytes (C, G, K) or in P2Y12‐expressing microglia (D, H, L). We regularly observed distinct clusters of GPER1 mRNA signal, which could not be attributed to any of the analyzed cell types (yellow arrows in D, L, for example). Thus, cell populations, which are not identified by the cell markers applied here, appear to express GPER1 mRNA in adult mouse hippocampus. These could be pericytes, which have recently been shown to express GPER1 in brain.^46^ gcl, granule cell layer; hil, hilus; so, stratum oriens; sp, stratum pyramidale; sr, stratum radiatum. Scale bar: 20 μm (Inset: 10 μm).

Generally, mRNA expression levels of estrogen receptors were markedly lower if compared with androgen receptors, consistent with the results of the qRT‐PCR analyses (Figure 2). Moreover, while ERα and GPER1 mRNA signal was still in an analyzable range (Figures 6, 7, 8), ERβ mRNA signal was overall barely detectable and we therefore abstained from performing quantitative cellular analyses for this receptor. For ERα mRNA, we noticed low abundance of signal in pyramidal cells of CA1 (Figures 6A and 7A) and CA3 (Figures 6A and 4E), as well as in granule cells and (presumed) hilar mossy cells in DG (Figures 6A and 7I). Low levels of ERα mRNA were on average also detected in interneurons (Figure 6A). However, here we noticed a remarkable variability, as a fraction of interneurons (~10%) expressed ERα mRNA quite robustly (AU: >8), whereas signal was almost undetectable (AU: <2) in most of the others (Figures 6A and 7B,F,J). Thus, in mouse hippocampus, ERα mRNA appears to be preferentially expressed in a specific subpopulation of GABAergic interneurons, as shown previously for rats.^45^ In contrast, virtually no ERα mRNA signal was detected in astrocytes (Figures 6A and 7C,G,K) and in microglia (Figures 6A and 7D,H,K), suggesting that these cell types do not substantially express ERα mRNA under physiological conditions. No subregional differences of ERα mRNA expression were observed.

For GPER1, substantial mRNA expression was detected in CamKII‐positive projection neurons, that is, in pyramidal cells of CA1 (Figures 6C and 8A) and CA3 (Figures 6C and 8E), and in granule cells and (presumed) hilar mossy cells of DG (Figures 6C and 8I). In contrast, GPER1 mRNA expression was overall low in interneurons (Figures 6C and 8B,F,J), and almost undetectable in astrocytes (Figures 6C and 8C,G,K) and in microglia (Figures 6C and 8D,H,L). As for ERα, no significant subregional differences were observed. Further of note: we regularly observed distinct clusters of GPER1 mRNA signal, which we could not attribute to any of the analyzed cell types (Figure 8D,J,L). This observation indicates that other than the here examined cell types may express GPER1 mRNA. A recent study suggests that these could be pericytes.^46^

As for the androgen receptors, comparing cellular expression data from male and female sections using Two‐Way ANOVA revealed sex‐related differences that partially supported the qRT‐PCR data. Thus, in accordance with the qRT‐PCR (Figure 2E), ERα mRNA signal was significantly stronger in sections from (proestrus) females compared with those from males. Cell‐type specific analyses suggested that this was mostly due to higher expression of ERα mRNA in female CamKII‐expressing projection neurons (Figure 6B). However, cellular GPER1 mRNA signal was also found to be significantly stronger in female sections, particularly in neurons (projection neurons and interneurons; Figure 6D), although this sex difference is not supported by the corresponding qRT‐PCR data (Figure 2D) and thus requires further substantiation.

## 4 DISCUSSION

In the brain, sex hormones do not only organize reproductive behaviors in the narrow sense, but also influence structural plasticity, as it is required, for example, for learning processes.^1^, ^2^ To understand these latter functions, reliable information about the expression patterns of the respective receptors is essential. However, previous attempts to determine these patterns were often hampered by the lack of reliable tools (e.g., specific antibodies; see Ref. [47]), thus in part revealing contradictory and ambiguous results. In this respect, the RNAscope in situ hybridization technique offers novel opportunities, as it allows detection of multiple targets with high sensitivity and specificity due to a novel probe design technology.^37^ We here used this technique for comparative analyses of the cellular mRNA expression of established androgen (AR, ZIP9) and estrogen receptors (ERα, ERβ, GPER1), and complemented these analyses with quantitative (total hippocampus) mRNA expression data from qRT‐PCR analyses. We further took potential effects of fluctuating sex hormones into consideration by analyzing data sex‐ and estrous cycle‐stage (“E2 high” vs. “E2 low”)‐specifically. Our major results are: (1) Androgen receptors are more abundantly expressed in adult mouse hippocampus than estrogen receptors, if the mRNA levels are considered. (2) Among the androgen receptors, AR mRNA is preferentially expressed in neurons of the CA1 and CA3 subfields, and lesser in those of the dentate gyrus. In astrocytes and microglia, only low levels of AR mRNA were detected. ZIP9 mRNA frequently co‐localizes with AR mRNA, but shows overall a more ubiquitous pattern of expression with no subregion preferences and including neurons as well as astrocytes and microglia. (3) Among the estrogen receptors, the expression of ERβ mRNA was so low that cellular expression could not reliably be determined. Due to its low level of expression, we regard ERβ unlikely to play major roles in adult mouse hippocampus. ERα and GPER1 mRNA were moderately expressed, but showed differential patterns of expression. Thus, while GPER1 mRNA was preferentially detected in CamKII‐positive (i.e., excitatory) neurons, ERα mRNA expression was most prominent in a subset of GABAergic interneurons. (4) Receptor mRNA expression appears to depend on sex (ERα) and can be subject to regulation by fluctuating sex hormones during the estrous cycle (ZIP9 mRNA; potentially also GPER1 mRNA, but evidence for a dependency of GPER1 mRNA expression on sex or cycle stage derives only from the cellular RNAscope data, which alone do not provide sufficient statistical stringency to draw such a conclusion).

Androgens are the predominant male sex hormones, but they exert important physiological effects also in females.^48^ In fact, serum testosterone levels in women are higher than estrogen levels most of the time (except during the preovulatory and midluteal phase of the menstrual cycle^48^). In the brain, however, testosterone is largely metabolized into E2, the most potent estrogen, by P450 aromatase,^49^ or into dihydrotestosterone (DHT), the most potent androgen, by 5α‐reductase.^50^ Both enzymes are substantially expressed in the adult rodent hippocampus,^51^, ^52^, ^53^, ^54^ generating DHT and E2 (DHT >> E2) intrinsically in roughly equal amounts in both sexes.^55^ This fact may account for the overall similar expression levels (except for ERα) of androgen and estrogen receptors in both sexes, while the preponderance of DHT could explain the stronger mRNA expression of androgen relative to estrogen receptors (here one needs to keep in mind, however, that mRNA levels may not strictly correlate with protein levels, as mRNA stability or translation rates could differ). The activities of aromatase and 5α‐reductase are further subject to regulation via the hypothalamic–pituitary–gonadal (HPG) axis.^56^ Thus, while the hippocampal functions of androgens and estrogens do not necessarily require peripheral supply of the respective hormones,^57^, ^58^, ^59^ various ways exist how these functions can be adapted to acute organismic requirements, which differ between males and females throughout life (e.g., during the estrous cycle).

The classical androgen receptor AR is considered a cytosol‐based receptor that translocates to the nucleus as a transcription factor upon ligand binding.^5^, ^6^, ^7^ Hippocampal AR has been detected by in situ hybridization or immunohistochemistry in adult rodents preferentially in pyramidal cells of CA1 and CA3 (with decreasing tendency), but less in DG granule cells.^26^, ^30^, ^31^, ^32^, ^33^, ^34^, ^35^, ^36^, ^60^ Our data largely confirm these previous findings. We further found AR expression in GABAergic interneurons,^61^ but cannot confirm substantial expression in astrocytes.^36^, ^62^, ^63^ However, as for ERs (see below), it is considerable that AR gets upregulated in astrocytes after hippocampal injury.^64^ Consistent with its pattern of expression, reported AR functions include modulation of glutamatergic transmission preferentially in CA1^65^, ^66^, ^67^, ^68^ and in CA3,^69^ which may impact performances in specific learning tasks.^66^, ^68^, ^70^ Effects of androgens on neurogenesis in DG have also been reported; however, these were mostly observed in young rodents, but not in adult rodents.^71^

Although androgen signaling is generally thought to involve AR‐mediated gene regulation, some rapid biological responses to androgenic stimulation suggest that non‐genomic pathways exist as well. While these could include non‐genomic actions of AR itself,^59^, ^72^, ^73^ insensitivity of some effects to AR inhibitors suggested that additional androgen receptors exist which are attached to the plasma membrane and connected to cellular signaling cascades.^61^, ^74^, ^75^ Several candidates have been identified,^9^ including a splice variant of AR (AR45) which is localized to plasma membrane lipid rafts and apparently functional in the hippocampus.^76^ Here we focused on determining the hippocampal expression of ZIP9, a G‐protein (Gnα11)‐coupled membrane protein, unrelated to AR and belonging to the Zrt‐, Irt‐like Protein (ZIP) Zn^2+^ transporter family which increases intracellular free Zn^2+^ levels by facilitating Zn^2+^ uptake from the extracellular fluid or releasing it from intracellular stores,^77^ but also binds androgens, although with markedly lower affinity than AR.^78^ Zn^2+^ regulates the expression of numerous genes via transcription factors containing Zn^2+^‐binding motifs (“zinc finger”^79^) and is a cofactor for over 50 metabolic enzymes.^80^ In the nervous system, additional functions apply, as Zn^2+^ can act as an allosteric modulator of synaptic receptors.^81^ Therefore, a complex Zn^2+^ transporter system tightly regulates intra‐ and extracellular Zn^2+^ levels within narrow limits.^77^ The hippocampus is a brain region with remarkably high Zn^2+^ content, showing the highest concentrations in the mossy fiber system and lower, but still substantial, levels in the strata oriens and radiatum of CA1 and CA3.^82^ At glutamatergic synapses, cytoplasmic Zn^2+^ is sequestered into round presynaptic vesicles via Zn^2+^ transporter ZnT3 and is released together with glutamate into the synaptic cleft.^82^ Postsynaptically, its major function appears to be a dampening of NMDA receptor‐mediated excitation.^83^ The abundant and ubiquitous expression of ZIP9 mRNA in all types of hippocampal cells, as shown here, suggests that ZIP9 could have an important part in re‐uptaking the synaptically released Zn^2+^ from the extracellular space and thus rendering it available for a new turn of release. In addition, by coupling with G‐proteins, ZIP9 appears to be able to transduce androgen signaling via second messenger pathways.^84^ The functions of this signaling in the brain yet remain to be elucidated. It was recently shown, however, that DHT‐mediated ZIP9 signaling increases the expression of synaptic proteins, such as PSD95, drebrin, or synaptophysin,^10^ and may thus strengthen AR‐mediated plasticity in hippocampal neurons,^69^ in which AR and ZIP9 mRNA are regularly co‐expressed, as shown here. Further, androgen binding to ZIP9 may increase the activity of the transporter,^84^ promoting the re‐uptake of Zn^2+^ that has been released due to AR‐stimulated glutamatergic activity.^66^, ^67^ Thus, AR and ZIP9 appear to mutually support each other. In this context, it is noteworthy that our data indicate a down‐regulation of hippocampal ZIP9 mRNA during phases of the estrous cycle, at which E2 levels are high (in this respect differing from ovarian ZIP9^78^). This implies that ZIP9‐mediated Zn^2+^‐uptake could be less effective, for example, in proestrus, potentially resulting in lesser availability of Zn^2+^ at glutamatergic synapses. In accordance with this observation, a down‐regulation of ZnT3 by E2 in the mossy fibers has previously been reported.^85^ In essence, both androgens and estrogens appear to affect the Zn^2+^ metabolism in the hippocampus and may thus critically influence excitability in the hippocampal network.

For the estrogen receptors, our data indicate substantial mRNA expression for GPER1 and a less abundant expression for ERα, whereas ERβ mRNA was virtually undetectable. These observations are in agreement with the findings from a recent study using droplet digital PCR to determine estrogen receptor profiles in adult rat hippocampus. These authors found a greater than two‐fold expression of GPER1 relative to ERα mRNA, and only very low levels of ERβ mRNA.^86^ However, while the ERα expression pattern, as shown here, was also largely found by earlier immunohistochemistry^13^, ^17^, ^20^, ^21^, ^22^ and in situ hybridization studies,^27^, ^28^ available information on ERβ is partially conflicting, as several studies reported substantial levels of ERβ mRNA^28^, ^29^ (but see^27^) or protein^11^, ^14^, ^17^, ^18^, ^21^ in adult hippocampus. In light of more recent studies using ERβ‐EGFP transgenic mice (demonstrating a developmental decrease of hippocampal ERβ^25^) or droplet digital PCR (see above^86^), we consider it very likely that our data reflect adult mouse hippocampal ERβ mRNA expression correctly, suggesting that ERβ plays a minor role in adult hippocampus. The reasons for the abovementioned discrepancies are not fully resolved, but may in part (i.e., for immunohistochemistry) result from unsatisfactory antibody specificity.^47^ In addition, the above cited studies often used female rodents which had been ovariectomized (e.g., Ref. [28]). Ovariectomy, however, disrupts the balancing of sex steroid hormones via the HPG axis, which could result in unphysiological changes of ER expression.^56^, ^87^

Our data further suggest differential roles for GPER1 and ERα, as these receptors are preferentially expressed in different cell types. For ERα, which has a stronger affinity to E2 compared with GPER1,^88^ highest mRNA expression levels were observed in a subset of GABAergic interneurons, while expression in glutamatergic neurons was comparably low. These observations are in accordance with the findings by Orikasa et al.^27^ and Hart et al.,^13^ who also found ERα to be preferentially expressed in a subpopulation (5%–14%) of GABAergic interneurons. Subsequent analyses identified these as cholecystokinin‐ (CCK), but not parvalbumin‐ (PV) positive basket cells.^45^ CCK‐positive basket cells fire at lower frequencies than PV‐positive basket cells, show accommodation, and release GABA asynchronously.^89^, ^90^ They further receive serotonergic input from the median raphe and are thus in a position to integrate local and subcortical inputs, and to fine‐tune the activity of pyramidal cells accordingly.^91^ It is therefore reasonable to assume that estrogens modify such fine‐tuning via ERα that is specifically localized on CCK‐positive basket cells.^45^ Of note: as this function requires fast responses to E2, ERα is unlikely to mediate it through gene transcription, but appears to act directly on ERα‐responsive synaptic vesicles.^45^ In contrast, GPER1 mRNA was preferentially found in glutamatergic cells, concordant with previous reports.^12^, ^15^, ^16^, ^19^, ^23^ GPER1 functions in hippocampus have not yet been fully elucidated. However, available evidence suggests that GPER1‐mediated E2 signaling generally dampens hippocampal excitability (e.g., by down‐regulating AMPA receptors^40^) and may specifically affect excitability at temporoammonic path synapses.^19^

Besides neurons, astrocytes^92^ and microglia^93^ have been reported to respond to estrogens. It was therefore unexpected to us not to also detect substantial levels of estrogen receptor mRNA in astrocytes or microglia. However, the responses of astrocytes to E2 do not necessarily require the expression of astrocytic receptors, as E2 may impose effects also via interactions with neurons. Thus, E2 can stimulate the maturation of astrocytes by inducing GABA release from neurons.^94^ Similarly, E2 infusion into the medial raphe^95^ or into the septal region^96^ caused a reduction of astroglia processes in the hippocampus, mediated apparently via the hippocampal afferents originating from these areas. Astrocyte activation by E2 may, on the other hand, influence the functions of microglia (and vice versa^97^, ^98^). However, small amounts of GPER1^15^, ^23^ and ERα were detected in astrocytes^99^ and in microglia (ERα^100^), and changes in receptor expression are induced in astrocytes and microglia after brain injury.^93^, ^101^ Thus, both cell types are generally capable of expressing estrogen receptors, but apparently do not do this substantially under physiological conditions. Further, pericytes—a cell type not considered in this study—were recently shown to express GPER1.^46^ Pericytes comprise, together with endothelial cells and astrocytes, the microvasculature in the brain, which cooperatively forms the blood–brain barrier. Expression of GPER1 in these cells suggests that this estrogen receptor is involved in controlling the blood flow in brain capillaries.^46^ While this issue awaits further clarification, the expression of GPER1 in pericytes may explain the presence of a population of cells that prominently expresses GPER1 mRNA but could not be attributed to one of the examined cell types (Figure 8, yellow arrows).

## 5 CONCLUSIONS

In summary, our study comprises a comparative analysis of the quantitative and cellular expression of androgen and estrogen receptors in the adult mouse hippocampus. Using the highly sensitive RNAscope in situ hybridization technique, the study demonstrates for the first time the mRNA expression pattern of ZIP9, an androgen‐modulated Zn^2+^‐transporter, and expands information on the expression of receptor types that have already been studied previously (AR, ERα, ERβ, GPER1), thereby clarifying some discrepancies. To these discrepancies may have contributed that previous studies often used animals of only one sex or precluded the female estrous cycle (e.g., by ovariectomy), thus largely eliminating the influences of peripheral sex hormones. In order to circumvent these limitations, we here studied both sexes and subdivided the females into an “E2 low” experimental subgroup with presumed low E2 serum levels (metestrus, estrus) and an “E2 high” group, in which E2 serum levels are assumed to be rising (diestrus) or high (proestrus^38^, ^39^). With this approach, we identified sex differences (e.g., a higher expression of ERα mRNA in female mouse hippocampus) and found evidence for a cycle‐stage dependent expression of ZIP9 mRNA, which was down‐regulated at stages with presumed high E2 serum levels, both in the qRT‐PCR and RNAscope analysis. However, while it is thus tempting to conclude that E2 modulates the expression of ZIP9 mRNA, caution is justified because the mouse estrous cycle does not only comprise fluctuating serum E2 levels. During “E2 high” phases of the cycle, serum DHT levels are also comparably high, while progesterone levels are lowest.^39^ Both changes can impact hippocampal functions via a variety of mechanisms,^102^ including a modulation of neuronal 5α‐reductase, which not only converts testosterone to DHT but also progesterone to allopregnanolone, a metabolite that enhances GABAergic inhibition, thus influencing hippocampal network excitability,^103^ which may in turn affect the expression of the sex hormone receptors. Therefore, more work is clearly necessary to unravel the factors and mechanisms that eventually cause specific changes of the transcriptome during the female estrous cycle. Generally, however, our results underline the necessity to consider sex and the female estrous cycle as influencing factors if one wants to understand the functions of sex hormone receptors.

## AUTHOR CONTRIBUTIONS

**Malte Schöbe:** Methodology; data curation; formal analysis. **Bianka Brunne:** Methodology; supervision. **Roland A. Bender:** Conceptualization; funding acquisition; writing – original draft; methodology; supervision; formal analysis.

## FUNDING INFORMATION

This study was financially supported by the Deutsche Forschungsgemeinschaft (DFG; RAB: Be4107/3‐1).

## CONFLICT OF INTEREST STATEMENT

The authors declare no conflicts of interest.

## PEER REVIEW

The peer review history for this article is available at https://www.webofscience.com/api/gateway/wos/peer-review/10.1111/jne.70086.

## Data Availability

The data that support the findings of this study are available on request from the corresponding author. The data are not publicly available due to privacy or ethical restrictions.
